# ECG differences and ECG predictors in patients presenting with ST segment elevation due to myocardial infarction versus takotsubo syndrome

**DOI:** 10.1016/j.ijcha.2022.101047

**Published:** 2022-05-06

**Authors:** Rickard Zeijlon, Jasmina Chamat, Vina Le, Johan Wågerman, Israa Enabtawi, Sandeep Jha, Mohammed Munir Mohammed, Aaron Shekka Espinosa, Oskar Angerås, Truls Råmunddal, Elmir Omerovic, Björn Redfors

**Affiliations:** a Department of Cardiology, Sahlgrenska University Hospital/S, Gothenburg, Sweden; b Department of Internal Medicine, Sahlgrenska University Hospital/S, Gothenburg, Sweden; c Wallenberg laboratory, Institute of Medicine, Sahlgrenska Academy, University of Gothenburg, Sweden; d Department of Cardiology, Sahlgrenska University Hospital/Ö, Gothenburg, Sweden; e Department of Internal Medicine, Kungälvs Hospital, Kungälv, Sweden; f Department of Cardiology, Norra Älvsborgs Länssjukhus, Trollhättan, Sweden; g Clinical Trial Center, Cardiovascular Research Foundation, NY, USA; h Department of Cardiology, New York-Presbyterian Hospital/Columbia University Medical Center, New York, USA; i Wallenberg Center for Molecular and Translational Medicine, University of Gothenburg, Gothenburg, Sweden

**Keywords:** Takotsubo syndrome, ST-elevation myocardial infarction, ECG, Arrhythmia

## Abstract

**Background:**

Previous studies comparing electrocardiogram (ECG) in Takotsubo syndrome (TS) versus ST elevation myocardial infarction (STEMI) included TS patients without ST elevation, did not consider the culprit lesion in STEMI or had groups that were unbalanced regarding sex and age. Accounting for these factors, we sought to conduct a more reliable comparison of ECG in TS with ST-elevation (STE-TS) versus STEMI. The secondary aim was to investigate if ST segment changes, T wave inversion or prolonged QT interval predicted ventricular arrhythmia or death in STE-TS and STEMI.

**Methods:**

All STE-TS patients who presented at Sahlgrenska University Hospital between 2008 and 2019 were matched by sex and age to STEMI patients. STEMI patients were subcategorized according to whether or not the culprit lesion was located in the left anterior descending artery (LAD). Baseline characteristics, in‐hospital outcomes and admission ECGs were analyzed.

**Results:**

104 STE-TS patients were sex- and age-matched with 274 STEMI patients (113 LAD-STEMI, 161 non-LAD STEMI). Admission ECG in STE-TS was more similar to LAD STEMI than non-LAD STEMI. Reciprocal ST depression was less common in STE-TS (7/104, 6.7%) compared with STEMI (112/274, 41%; p= < 0.001). The sum of all ST elevations and the sum of all ST-deviations predicted life-threatening ventricular arrhythmia (LTVA) or death in LAD STEMI but not in STE-TS.

**Conclusions:**

In conclusion, admission ECG in STE-TS was similar to LAD STEMI but reciprocal ST depression was less common in STE-TS compared with STEMI overall. ST segment changes predicted LTVA or death in STEMI but not in STE-TS.

## Highlights

• Takotsubo syndrome and myocardial infarction can present with ST segment elevation.
• ECG in Takotsubo resembles left anterior descending artery myocardial infarction.
• Ventricular arrhythmia or death occur in both Takotsubo and myocardial infarction.
• ST segment changes predict ventricular arrhythmia or death in myocardial infarction.
• ST segment changes do not predict ventricular arrhythmia in Takotsubo syndrome.

## Graphical abstract

**Figure:**
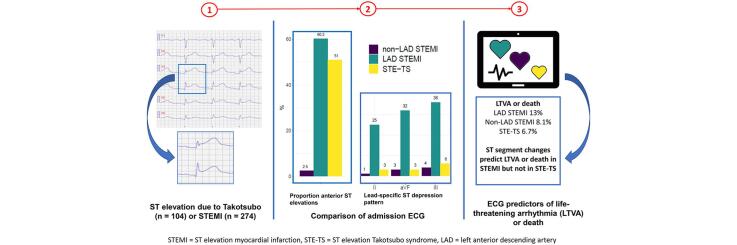

## 1 Introduction

Takotsubo syndrome (TS) and ST elevation myocardial infarction (STEMI) are acute cardiac conditions with similar initial symptoms, non-invasive test results and complications. Both conditions can present with ST elevation on electrocardiogram (ECG) and are associated with life-threatening ventricular arrhythmia and death [1], [2]. However, the pathophysiology is different. Whereas STEMI is caused by an acute coronary occlusion (requiring immediate percutaneous coronary intervention (PCI) to limit the extent of myocardial injury), TS is characterized by transient left ventricular dysfunction caused by emotional or physical stress and is self-limiting without PCI. [1], [2], [3], [4].

The initial ECG is similar in TS and STEMI, and about 45% of TS patients present with ST elevation [5], [6]. In both conditions, T wave inversion develops whereas QT interval prolongation is more typical for the temporal ECG development in TS [3], [6]. Several methods, based on a variety of ECG changes, have been suggested to distinguish TS from STEMI [6], [7]. TS presenting with ST elevation (STE-TS) is especially challenging in the differential diagnosis against STEMI and none of the proposed methods can distinguish TS from STEMI reliably enough to avoid coronary angiography.

The occurrence of ventricular arrhythmia or death in STE-TS versus STEMI has only been investigated in a few small cohorts [8], [9]. Furthermore, to what extent ECG predictors of outcome differs between STE-TS and STEMI is largely unknown.

Our primary aim was to conduct a detailed comparison of admission ECG changes in an age- and sex matched population of STE-TS and STEMI, with patients with STEMI further subdivided in those with culprit lesion in the left anterior descending artery (LAD) versus a non-LAD vessel. Our secondary aim was to investigate if ST segment changes, T wave inversion or long corrected QT interval (QTc) predicted ventricular arrhythmia or death in STE-TS or STEMI.

## 2 Methods

The study cohort consisted of patients with suspected TS and STEMI who were admitted to Sahlgrenska University Hospital between January 2008 and January 2019. Patients were identified using the Swedish Coronary Angiography and Angioplasty Registry (SCAAR). As previously described [10], medical charts were reviewed for all patients who presented with suspected TS during the study period, of whom 213 fulfilled the European Society of Cardiology (ESC) diagnostic criteria for TS [11]. All TS patients underwent coronary angiography to exclude acute coronary occlusion as the cause of cardiac dysfunction. Medical charts were also reviewed for all STEMI patients enrolled in the previously described cohort (n = 596) to confirm the diagnosis [10].

Exclusion criteria for all patients were pacemaker rhythm or left bundle branch block (LBBB) on admission; previous coronary artery bypass graft (CABG) surgery or not having ST elevation on admission ECG. In the STEMI cohort, patients with posterior STEMI were excluded. Each patient with STE-TS was then matched by sex and age with 1 to 3 patients from the STEMI cohort. STEMI patients were subdivided into STEMI with left anterior descending artery (LAD) occlusion and STEMI with non-LAD occlusion.

Admission ECG was available for all patients. For STEMI patients, primary percutaneous intervention (PCI) was performed within a median of 53 (IQR 26–91) minutes from ECG diagnosis. All 12-lead ECGs were recorded at a paper speed of 50 mm/s and an amplification of 10 mm/mV. ST segment deviation was measured manually at the J-point from the isoelectric line to the nearest 0.5 mm. T wave and Q wave amplitudes were measured manually from the isoelectric line to peak or nadir to the nearest 0.5 mm. Electronically derived values for heartrate, PR interval, QRS duration, QRS axis, T wave axis and QT time were chosen if assessed manually as correct. The corrected QT interval (QTc) was calculated using Bazzet’s formula.

All patients were monitored by telemetry during their entire hospitalization. Detailed information of arrhythmias was documented by thorough review of the telemetry recordings 3 times per day as part of routine clinical care. Information regarding admission clinical variables, ongoing medical treatment, acute heart failure, left ventricular ejection fraction (LVEF) and in-hospital arrhythmias were collected from patient charts. Information on co-morbidities was obtained from SCAAR.

LAD STEMI was defined as STEMI with culprit lesion in LAD or any of its branches; and non-LAD STEMI was defined as STEMI with culprit lesion in the right coronary artery (RCA) or left circumflex artery (LCx), or any of their branches. Acute heart failure (AHF) was defined as Killip class ≥ 2 and cardiogenic shock (CS) as Killip class 4. All definitions related to ECG or arrhythmia are summarized in Supplementary Table 1.

Within our secondary aim (ECG predictors of ventricular arrhythmia or death) the primary endpoint was the composite of life-threatening ventricular arrhythmia (LTVA) or death within 72 h after hospital admission. The secondary endpoint (within our secondary aim) was the composite of any sustained or non-sustained ventricular tachycardia or ventricular fibrillation (any VT/VF) or death within 72 h.

### 2.1 Statistical analysis

Variables are presented as mean ± standard deviations, median and interquartile range, or percentages for categorical variables. Categorical variables were compared using Chi-Square test or Fischer’s Exact test and continuous variables were compared using ANOVA for normally distributed variables and Kruskal-Wallis Test for non-normally distributed variables. Univariable and multivariable logistic regression was used to assess the unadjusted and adjusted association between ECG changes and outcomes. All statistical analyses were performed using SPSS (IBM) version 27 and all figures were created using R-studio version 1.4.1103 (*gglot, Tidyverse package in R*). The level of significance was set at p < 0.05.

The study was conducted in accordance with the Declaration of Helsinki and was approved by the Swedish Ethical Review Authority (registration number 2020–01569) and individual consent for this retrospective analysis was waived.

## 3 Results

### 3.1 Baseline characteristics

The study cohort consisted of 104 patients with STE-TS and 274 patients with STEMI, of whom 113 patients had LAD STEMI and 161 patients had non-LAD STEMI. Most baseline characteristics were similar between the groups *(*Table 1*)*, but STE-TS-patients had a lower proportion of diabetes and had lower BMI compared to patients with STEMI. STE-TS-patients were also less frequently treated with beta-blockers or diuretics than STEMI-patients, and fewer patients with STE-TS or LAD STEMI smoked compared with non-LAD STEMI. Presenting with angina was less common, whereas presenting with dyspnea or syncope was more common, in STE-TS versus STEMI. Heart rate was higher in STE-TS and LAD STEMI than in non-LAD STEMI. STE-TS-patients presented with the lowest LVEF, followed by LAD STEMI and non-LAD STEMI respectively. Consistent with this, AHF on admission was more common in STE-TS and LAD STEMI compared with non-LAD STEMI.

**Table 1:** Baseline characteristics and presenting symptoms.

	STEMI N = 274		
Variable	LAD N = 113	Non-LAD N = 161	STE-TS N = 104
*Demographics*			
Age (years)	71 ± 14	68 ± 13	69 ± 13
Female sex % (n/N)	89% (100/113)	89% (143/161)	89% (93/104)
BMI	27 ± 4.5	27 ± 5.6	24 ± 4.4
Diabetes	12% (13/111)	15% (24/157)	1% (1/103)
Current smoking	21% (20/94)	39% (54/140)	21% (19/91)
Hypertension	51% (55/107)	44% (59/156)	38% (39/102)
Hyperlipidemia	17% (18/106)	15% (23/151)	12% (12/101)
Previous myocardial infarction	6.4% (7/109)	8.2% (13/159)	4.8% (5/103)
Previous PCI	4.4% (5/113)	5.6% (9/161)	2.9% (3/104)
Hospitalized ≥ 72 h after index event* % (n/N)	88% (92/105)	79% (122/155)	80% (79/99)
*Presenting symptoms and signs*			
Heart rate (beats per minute)	83 (69–99)	68 (55–82)	87 (76–102)
Systolic blood pressure (mmHg)	138 ± 24	136 ± 30	138 ± 26
Diastolic blood pressure (mmHg)	85 ± 17	80 ± 19	83 ± 17
Oxygen saturation (%)	95 (93–98)	97 (95–99)	95 (92–97)
Angina % (n/N)	96% (102/113)	90% (154/161)	68% (71/104)
Dyspnea	13% (15/113)	10% (16/161)	34% (35/104)
Syncope	4.3% (5/113)	6.2% (10/161)	12% (12/104)
Killip Class ≥ 2	27% (30/113)	16% (26/161)	29% (30/102)
Killip Class 4	4.4% (5/113)	6.8% (11/161)	2.9% (3/102)
Femoral access	29% (33/113)	34% (55/161)	30% (31/104)
LVEF on admission %	45 (35–50)	55 (45–60)	40 (35–45)
Typical apical takotsubo	NA	NA	94% (98/104)
Emotional trigger takotsubo†	NA	NA	35% (36/104)
Physical trigger takotsubo	NA	NA	22% (23/104)
*Home medications % (n/N)*			
Beta-blockers	22% (25/113)	26% (42/161)	13% (13/104)
ACEI/ARB	21% (24/113)	24% (39/161)	23% (24/104)
Mineralocorticoid antagonist	0% (0/113)	3.1% (5/161)	1.9% (2/104)
Diuretics	15% (17/113)	19% (30/160)	7.7% (8/104)
Calcium antagonists	16% (18/113)	14% (23/161)	9.6% (10/104)
Aspirin	13% (15/113)	16% (25/161)	14% (15/104)
P2Y12 antagonist	1.8% (2/113)	3.7% (6/161)	1.0% (1/104)
OAC/Warfarin	4.4% (5/113)	3.7% (6/161)	1.9% (2/104)
Statins	12% (14/113)	17% (27/161)	9.6% (10/104)
Antiarrhythmic agents (non-beta blocker)	0% (0/113)	0% (0/161)	0% (0/104)

ACEI/ARB = angiotensin-converting enzyme inhibitors or angiotensin receptor blockers; BMI = body mass index; LAD = left anterior descending artery; LVEF = left ventricular ejection fraction; NA = not applicable; OAC = oral anticoagulants; PCI = percutaneous coronary intervention; STEMI = ST elevation myocardial infarction; STE-TS = ST elevation Takotsubo syndrome.

* Patients who survived 72 h.

† For the remaining TS-patients no identified trigger.

### 3.2 Admission ECG

**Fig. 1:**
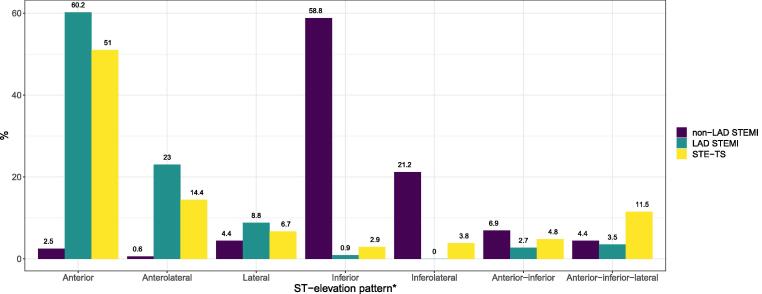
**ST elevation pattern on admission.** *All presented ST elevation patterns are mutually exclusive. Anterior = ST elevation in 2 consecutive leads in V1-V4; anterior-inferior = ST elevation in 2 consecutive leads in V1-V4 and II-aVF or aVF-III; anterior-inferior-lateral = ST elevation in 2 consecutive leads in V1-V4 and II-aVF or aVF-III and V5-V6 or I-aVL; anterolateral = ST elevation in at least 2 consecutive leads in V1-V4 and V5-V6 or I-aVL; inferior = ST elevation in leads II-aVF or aVF-III; inferolateral = ST elevation in leads II-aVF or aVF-III and I-aVL or V5-V6; lateral = ST elevation in V5-V6 or I-aVL. LAD = left anterior descending artery; STEMI = ST elevation myocardial infarction; STE-TS = ST elevation takotsubo syndrome.

**Fig. 2:**
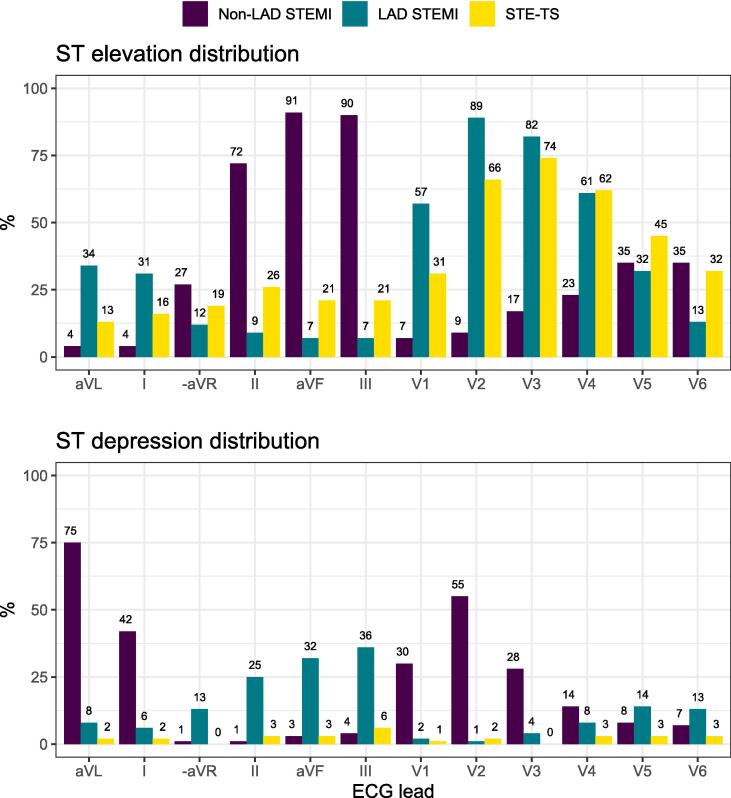
**ST elevation and ST depression distribution on admission.** LAD = left anterior descending artery; STEMI = ST elevation myocardial infarction; STE-TS = ST elevation takotsubo syndrome.

The ST elevation pattern in STE-TS resembled LAD STEMI more than non-LAD STEMI, with ST elevation in at least 2 consecutive anterior leads in 82% of STE-TS patients *(*Fig. 1*)*. Although most ECG changes were similar in STE-TS and LAD STEMI, it was less common for patients with STE-TS than LAD STEMI to present with ST elevation with reciprocal ST depression or ST depression per se. When comparing STE-TS to *non-LAD STEMI*, most ECG findings were significantly different. Lead-specifically, ST elevation in the anterior leads V2-V5 was more common in STE-TS than ST elevation in the inferior leads II, aVF and III. Thus, the ST elevation distribution in STE-TS was considerably more similar to LAD than non-LAD STEMI. QTc prolongation was more common in STE-TS compared with non-LAD STEMI but similar to LAD STEMI, and T wave inversion was less common in STE-TS compared with STEMI overall. All ECG changes on admission are summarized in Table 2 and lead-specific patterns of ST elevation, ST depression and T wave inversion are summarized in Fig. 2, Supplementary figure 2 *and* Supplementary Table 2 .

**Table 2:** ECG on admission.

	STEMI N = 274			p-values			
Variable	LAD N = 113	Non-LAD N = 161	STE-TS N = 104	LAD vs non-LAD	LAD vs STE-TS	Non-LAD vs STE-TS	STEMI all vs STE-TS
Rhythm % (n/N)							
Sinus	93% (105/113)	87% (140/161)	96% (100/104)	0.11	0.30	0.012	0.038
Atrial fibrillation or flutter	5.3% (6/113)	5.6% (9/161)	3.8% (4/104)	0.92	0.75	0.52	0.52
AV nodal	0.9% (1/113)	6.2% (10/161)	0% (0/104)	0.030	>0.99	0.0073	0.039
Other	0.9% (1/113)	1.2% (2/161)	0% (0/104)	>0.99	>0.99	0.52	0.56
PR interval (milliseconds)	165 (146–186)	164 (150–194)	156 (140–172)	0.50	0.0058	<0.001	<0.001
AV conduction % (n/N)							
Normal	95% (103/108)	87% (124/142)	99% (99/100)	0.029	0.21	<0.001	0.0061
AV block 1	3.7% (4/108)	9.2% (13/142)	1.0% (1/100)	0.090	0.37	0.0075	0.026
AV block 2a	0% (0/108)	0% (0/142)	0% (0/100)	N/A	N/A	N/A	N/A
AV block 2b	0% (0/113)	0% (0/161)	0% (0/100)	N/A	N/A	N/A	N/A
AV block 3	0.9% (1/108)	3.5% (5/142)	0% (0/100)	0.24	>0.99	0.079	0.19
QRS duration (milliseconds)	90 (80–100)	92 (84–100)	88 (83–98)	0.082	0.90	0.059	0.20
QRS axis (degrees)	6.0 (–32–52)	51 (14–73)	25 (-27–68)	<0.001	0.040	0.0038	0.42
T wave axis	48 (2.5–81)	88 (62–98)	69 (53–80)	<0.001	0.0011	<0.001	0.42
QTc interval (milliseconds)	444 (420–463)	431 (415–448)	451 (424–472)	0.0036	0.13	<0.001	<0.001
Long QTc* % (n/N)	31% (34/111)	21% (32/156)	39% (41/104)	0.059	0.18	<0.001	0.0050
QTc > 500 ms	6.3% (7/111)	1.9% (3/156)	7.7% (8/104)	0.099	0.69	0.030	0.11
Q wave pathology	31% (35/113)	26% (41/161)	36% (37/104)	0.32	0.47	0.078	0.14
Fragmented QRS	49% (55/113)	49% (79/161)	42% (44/104)	0.95	0.35	0.28	0.25
Low voltage QRS	17% (19/113)	6.2% (10/161)	22% (23/104)	0.0050	0.32	<0.001	0.0037
ST elevation with reciprocal ST depression	24% (27/113)	53% (85/161)	6.7% (7/104)	<0.001	<0.001	<0.001	<0.001
ST depression	37% (42/113)	65% (105/161)	9.6% (10/104)	<0.001	<0.001	<0.001	<0.001
T wave inversion	52% (59/113)	86% (139/161)	39% (41/104)	<0.001	0.059	<0.001	<0.001
ST elevation pattern on admission							
Anterior†	60% (68/113)	2.5% (4/160)	51% (53/104)	<0.001	0.17	<0.001	<0.001
Lateral‡	8.8% (10/113)	4.4% (7/160)	6.7% (7/104)	0.13	0.56	0.40	0.86
Inferior§	0.9% (1/113)	59% (94/160)	2.9% (3/104)	<0.001	0.35	<0.001	<0.001
Anterolateral||	23% (26/113)	0.6% (1/160)	14% (15/104)	<0.001	0.11	<0.001	0.21
Inferolateral#	0% (0/113)	21% (34/160)	3.8% (4/104)	<0.001	0.051	<0.001	0.013
Anterior-inferior**	2.7% (3/113)	6.9% (11/160)	4.8% (5/104)	0.17	0.49	0.49	0.90
Anterior-inferior-lateral††	3.5% (4/113)	4.4% (7/160)	12% (12/104)	>0.99	0.024	0.028	0.0065
Other‡‡	0.9% (1/113)	1.3% (2/160)	4.8% (5/104)	>0.99	0.11	0.12	0.039

AV = atrio-ventricular; ECG = electrocardiography; LAD = left anterior descending artery; N/A = not applicable; STE = ST elevation; STEMI = ST elevation myocardial infarction; STE-TS = ST elevation takotsubo syndrome.

* Long QTc > 440 ms for men, > 460 ms for women.

† ST elevation in two consecutive leads in V1-V4.

‡ ST elevation in V5-V6 or I-aVL.

§ ST elevation in leads II-aVF or aVF-III.

|| ST elevation in at least two consecutive leads in V1-V4 and V5-V6 or I-aVL.

# ST elevation in leads II-aVF or aVF-III and I-aVL or V5-V6.

** ST elevation in two consecutive leads in V1-V4 and II-aVF or aVF-III.

†† ST elevation in two consecutive leads in V1-V4 and II-aVF or aVF-III and V5-V6 or I-aVL.

‡‡ Other pattern not fitting any of the stated ST elevation patterns.

Concave ST elevation was more common in STE-TS compared with STEMI, except in the “high lateral” leads (aVL and I). The difference was most pronounced in the “low lateral” leads (V5 or V6) where a majority of patients with STE-TS had concave ST-elevation, compared with a minority of patients with STEMI (65% versus 22%, p < 0.001) (Supplementary Figure 2*).*

### 3.3 ECG predictors of ventricular arrhythmia or death

Of the 378 patients, 19 died and 20 suffered from LTVA within 72 h from hospitalization. There were no significant differences between STE-TS, LAD or non-LAD STEMI in the occurrence of the composite of LTVA or death within 72 h *(*Table 3*).* The crude mortality within 72 h was similar across the 3 groups, while the occurrence of LTVA was numerically lower in STE-TS than STEMI overall (1.9% vs 6.6%, p = 0.072). The incidence of any VT/VF or death was substantially lower in STE-TS compared to STEMI, driven by a lower incidence of VT/VF.

**Table 3:** Complications.

	STEMI N = 274			p-values			
Variable	LAD N = 113	Non-LAD N = 161	STE-TS N = 104	LAD vs Non-LAD	LAD vs STE-TS	Non-LAD vs STE-TS	STEMI all vs STE-TS
LTVA or death 72 h	13% (15/113)	8.1% (13/161)	6.7% (7/104)	0.16	0.11	0.69	0.30
LTVA	7.1% (8/113)	6.2% (10/161)	1.9% (2/104)	0.78	0.10	0.13	0.072
Sustained VT	2.7% (3/113)	3.1% (5/161)	1.0% (1/104)	>0.99	0.62	0.41	0.46
VF	5.3% (6/113)	5.0% (8/161)	1.0% (1/104)	0.90	0.12	0.094	0.078
Death	7.1% (8/113)	3.7% (6/161)	4.8% (5/104)	0.22	0.48	0.76	>0.99
VT/VF or death 72 h	40% (45/113)	46% (74/161)	11% (11/104)	0.31	<0.001	<0.001	<0.001
VT/VF	40% (45/113)	46% (74/161)	11% (11/104)	0.31	<0.001	<0.001	<0.001
Any VT*	36% (41/113)	44% (71/161)	9.6% (10/104)	0.19	<0.001	<0.001	<0.001

LAD = left anterior descending artery; LTVA = life threatening ventricular arrhythmia; STEMI = ST elevation myocardial infarction; STE-TS = ST elevation takotsubo syndrome; VF = ventricular fibrillation; VT = ventricular tachycardia.

* Any sustained or non-sustained ventricular tachycardia.

After multivariable adjustment for baseline risk factors, the sum of all ST elevations and the sum of all ST-deviations were independent predictors of LTVA or death among patients with LAD STEMI *(*Table 4*)*. Among patients with non-LAD STEMI, the sum of all ST deviations and the maximum single lead ST-elevation were independent predictors of LTVA or death. None of the investigated ECG changes predicted LTVA or death in STE-TS.

**Table 4:** Predictors of LTVA or death within 72 h in patients with STEMI (LAD and non-LAD) and STE-TS.

Variable	LAD N = 113		Non-LAD N = 161		STE-TS N = 104	
	OR (95 %CI)	p-value	OR (95 %CI)	p-value	OR (95 %CI)	p-value
Sum of all ST-elevations						
Univariable	1.07 (1.00 – 1–14)	0.040	1.07 (0.991 – 1.15)	0.084	0.996 (0.835 – 1.19)	0.96
Model A*	1.08 (1.01 – 1.15)	0.029	1.08 (0.995 – 1.16)	0.065	0.993 (0.828 – 1.19)	0.94
Model B†	1.08 (1.00 – 1.16)	0.043	1.08 (0.995 – 1.17)	0.067	0.993 (0.827 – 1.19)	0.94
Sum of all ST-deviations						
Univariable	1.07 (1.01 – 1.13)	0.015	1.08 (1.02 – 1.14)	0.0080	1.01 (0.855 – 1.20)	0.90
Model A	1.08 (1.02 – 1.14)	0.012	1.09 (1.02 – 1.15)	0.0066	1.01 (0.851 – 1.20)	0.90
Model B	1.11 (1.03 – 1.19)	0.0072	1.09 (1.02 – 1.16)	0.0086	0.941 (0.846 – 1.20)	0.94
Maximum single-lead ST-elevation						
Univariable	1.28 (0.999 – 1.64)	0.051	1.45 (1.07 – 1.97)	0.017	0.576 (0.209 – 1.59)	0.29
Model A	1.31 (1.01 – 1.70)	0.040	1.64 (1.16 – 2.31)	0.0046	0.547 (0.189 – 1.59)	0.27
Model B	1.30 (0.983 – 1.73)	0.066	1.63 (1.15 – 2.32)	0.0067	0.561 (0.192 – 1.64)	0.29
ST-elevation with reciprocal ST-depression						
Univariable	1.73 (0.534 – 5.59)	0.36	2.13 (0.629 – 7.23)	0.22	2.53 (0.261 – 24.5)	0.42
Model A	1.65 (0.503 – 5.39)	0.41	2.11 (0.617 – 7.18)	0.23	0.426 (0.252 – 26.2)	0.43
Model B	2.49 (0.701 – 8.87)	0.16	1.78 (0.510 – 6.24)	0.37	2.16 (0.204 – 22.9)	0.52
T wave inversion						
Univariable	1.44 (0.476 – 4.35)	0.52	NA	NA	0.238 (0.0275 – 2.05)	0.19
Model A	1.14 (0.469 – 4.31)	0.53	NA	NA	0.210 (0.0233 – 1.89)	0.16
Model B	0.913 (0.274 – 3.05)	0.88	NA	NA	0.217 (0.0239 – 1.96)	0.17
Long QTc‡						
Univariable	1.16 (0.363 – 3.68)	0.81	1.83 (0.524 – 6.36)	0.35	4.24 (0.781 – 23.0)	0.094
Model A	1.15 (0.358 – 3.71)	0.81	1.90 (0.535 – 6.72)	0.32	4.10 (0.746 – 22.6)	0.11
Model B	1.75 (0.477 – 6.39)	0.40	1.53 (0.378 – 6.15)	0.55	3.82 (0.684 – 21.4)	0.13

LAD = left anterior descending artery; LTVA = life threatening ventricular arrhythmia; NA = not applicable because of zero events in one of the categories;

STEMI = ST elevation myocardial infarction; STE-TS = ST elevation takotsubo syndrome.

* Adjusted for age and sex.

† adjusted for age, sex, diabetes and previous myocardial infarction.

‡ Long QTc > 440 ms for men, > 460 ms for women.

Independent predictors of *any* VT/VF or death within 72 h are presented in Supplementary Table 3. The sum of all ST elevation and the sum of all ST deviations were independent predictors of VT/VF or death in both LAD and non-LAD STEMI. The maximum single lead ST elevation and ST elevation with reciprocal ST depression were also independent predictors of VT/VF or death in non-LAD STEMI. Long QTc was associated with a lower risk of VT/VF or death in LAD STEMI. Among patients with STE-TS, T-wave inversion was associated with significantly lower risk of VT/VF or death after adjustment for age and sex, but this association was not significant after adjustment for other risk factors. No other ECG characteristics predicted the occurrence of VT/VF or death among patients with STE-TS.

## 4 Discussion

### 4.1 Admission ECG

Our main finding was that admission ECG in STE-TS was considerably more similar to STEMI with culprit lesion in LAD compared with a non-LAD vessel (LCx or RCA). Patients with STE-TS were less likely to present with reciprocal ST depression compared with STEMI, but we found no ECG criteria that could reliably differentiate between STE-TS and STEMI.

Although ECG in STE-TS and LAD STEMI was similar, this study adds novel aspects regarding ECG differences between the two conditions. The lead-specific ST elevation- and T wave inversion distribution was similar in STE-TS and LAD STEMI, however, the ST depression distribution was different. Almost 1 of 3 LAD STEMI patients presented with ST depression in inferior leads (II, aVF or III) whereas nearly 1 of 4 STE-TS patients presented with *ST elevation* in these leads. Furthermore, according to previous literature, non-ischemic conditions involving ST elevation present with *concave* ST elevation more often than ischemic conditions [12]. Interestingly, we found concave ST elevation to be considerably more common in STE-TS than STEMI. This was most pronounced in the “low lateral” leads (V5-V6), where concave ST elevation 4 times more common in STE-TS compared to STEMI.

Most previous studies comparing ECG in TS versus STEMI have been based on mixed populations of TS with and without ST elevation [13], [14], [15], [16], [17], [18], [19], [20], [21], and/or mixed populations of STEMI or non-STEMI [13], [15], [22], [23]. The previous studies investigating ECG in TS with ST elevation specifically versus STEMI did not match their cohorts by sex and did not discriminate between both anterior and non-anterior STEMI [24], [25], [26], [27]. Therefore, our study could provide a more clinically representative picture of the typical admission ECG pattern in STE-TS in relation to STEMI. Additionally, with respect to the exact localization and distribution of ST depressions, our findings are an important extension of the previous knowledge that the absence of *reciprocal* ST depression per se suggests STE-TS over STEMI [7], [17], [23], [25], [27].

The absence of reciprocal ST depression in STE-TS may be attributed to the absence of transmural ischemia which is believed to explain the reciprocal ST depression seen in STEMI. Also, the wall-motion abnormality seen in TS extends beyond the territory of a single coronary artery. This differs from the ischemic wall-motion abnormality seen in STEMI, where focal ischemia forms the basis for normal and abnormal wall-motion in electrically opposite parts of the heart [28]. In accordance with this, an extensive ST elevation pattern with a combined anterior, inferior and lateral ST elevation pattern was more common in STE-TS compared with STEMI in general and LAD STEMI in particular.

QTc-prolongation and T wave inversion have been suggested as more common, and pathological Q-waves as less common, in TS compared with STEMI [6], [15]. Although more common in STE-TS than STEMI, long QTc was present in 1 of 4 patients with STEMI in the present analysis. The proportion of T wave inversion in STE-TS was similar to previous studies [5], [6], however, T wave inversion on admission was more common in STEMI than in STE-TS. This finding, together with previous research showing QTc-prolongation in the subacute phase of TS (day 1–3, along with progressive T wave inversion) [29], point towards T wave inversion and QTc prolongation as sub-optimal markers for TS versus STEMI in the acute phase. Since ST elevation is most common in the earliest phase of TS [28], the patients in our TS cohort (with STE-TS exclusively) were probably all in an early phase of TS. We found similar rates of pathologic Q-waves in STE-TS versus STEMI which is probably also explained by STE-TS patients being in an early phase of TS. Transient pathologic Q-waves in TS has been attributed to reversible myocardial stunning, where most previous studies describe pathologic Q-waves in the early phase of TS with rapid reappearance of R-waves [24], [28].

### 4.2 ECG predictors of ventricular arrhythmia or death

Within 72 h, we found that the occurrence of LTVA was numerically lower, and the occurrence any VT/VF was considerably lower, in STE-TS compared with STEMI. However, there was no difference in the crude rate of death within 72 h. This is consistent with previous studies that have shown lower rates of ventricular arrhythmia or cardiac arrest [2], [10], [30], [31] in TS compared to STEMI but similar mortality [6]. A larger sample size may have been needed to reflect the true difference in occurrence of LTVA between STE-TS and STEMI in the present study.

The sum of all ST elevations and the sum of all ST deviations independently predicted LTVA or death in LAD STEMI, and the sum of all ST deviations and the maximum single lead ST elevation predicted LTVA or death in non-LAD STEMI. These findings are consistent with previous studies [32], [33], [34]. None of the investigated parameters predicted LTVA or death in STE-TS. However, T-wave inversion at presentation was associated with a lower risk of any VT/VF or death in STE-TS after adjusting for age and sex.

We previously demonstrated an association between T-wave-inversion at presentation and a lower risk of in-hospital VT/VF in TS [35]. In myocardial ischemia–reperfusion, T-wave inversion has been attributed to viable but sympathetically denervated myocardium and previous research have shown that sympathetic denervation can reduce ventricular arrhythmia in patients with structural heart disease [36], [37]. Interestingly, sympathetic denervation has also been demonstrated in association with stress induced left ventricular dysfunction [38]. Sympathetic denervation, in the absence of myocardial ischemia or necrosis, could hypothetically explain the lower rates of VT/VF observed in association with T-wave inversion in STE-TS in the present analysis.

The presence of an association between ST segment changes and LTVA our death in STEMI, and the absence of such an association in STE-TS, could be explained by the difference in pathophysiology between the two conditions. In STEMI, ST-elevation is related to a combination of myocardial stunning and transmural ischemia, where a higher degree of myocardial ischemia with larger infarct size is associated with poor outcome. In STE-TS, ST-elevation can be explained by isolated reversible myocardial stunning, where the absence of widespread ischemia probably explains the lack of association between ST segment changes and poor outcome. [28], [39], [40].

In the present study, long QTc was associated with a lower risk of VT/VF or death in LAD STEMI and a trend towards lower risk of VT/VF or death in STE-TS. As opposed to the acquired long QT-syndrome associated with higher frequency of ventricular arrhythmia in STEMI and TS [6], transient QTc-prolongation has previously been associated with stunned viable myocardium and smaller infarct size in anterior STEMI [41]. In TS, previous research has shown that long QTc was associated with ventricular arrhythmias after 48 h but not at admission [42]. The phenomenon with transient long QTc as a marker for stunned viable myocardium may explain our association between long QTc and lower risk of VT/VF or death in LAD STEMI and STE-TS.

### 4.3 Strengths and limitations

The main strengths of our ECG analysis compared to previous studies were the high detail level and the separate comparisons to both LAD and non-LAD STEMI. Other strengths were the relatively large cohort and the matching of patients with STE-TS vs STEMI by age and sex. This is important because TS is more common in postmenopausal women [4], [5] whereas STEMI is more common men. Also, men on average develop STEMI at a younger age compared to women [2], [43], [44]. Because of its retrospective nature, the study is limited by the fact that we were not able to able to obtain information that was not already in the patient’s medical chart. However, medical charts, ECGs and echocardiographic examinations were reviewed for all patients to validate the diagnosis of TS or STEMI, and clinical admission parameters, telemetry reports and complications were available for all patients. Because of multiple comparisons of ECG variables, the risk of multiplicity and statistical type I error must be considered when interpreting our results regarding ECG changes. However, the differences that are emphasized is this study were large. Lastly, our cohort was predominately female which makes our results mainly applicable to female patients with STE-TS and STEMI.

## 5 Conclusions

Admission ECG in STE-TS was more similar to STEMI with culprit lesion in LAD compared with a non-LAD vessel. Reciprocal ST depression was less common in STE-TS compared with both LAD and non-LAD STEMI. The magnitude of the deviation of the ST-segment from the isoelectric line was an independent predictor of LTVA or death within 72 h in STEMI while none of the investigated ECG changes predicted LTVA or death in STE-TS.

## Declaration of Competing Interest

The authors declare that they have no known competing financial interests or personal relationships that could have appeared to influence the work reported in this paper.
