# Supplementary material for: ECG differences and ECG predictors in patients presenting with ST segment elevation due to myocardial infarction versus takotsubo syndrome
Source: Int J Cardiol Heart Vasc. 2022 May 6;40:101047. doi: 10.1016/j.ijcha.2022.101047 (PMC9096129; doi:10.1016/j.ijcha.2022.101047)
Supplement: Supplementary Figure 1 [file mmc1.pdf]

Supplementary Figure 1. T-inversion distribution on admission

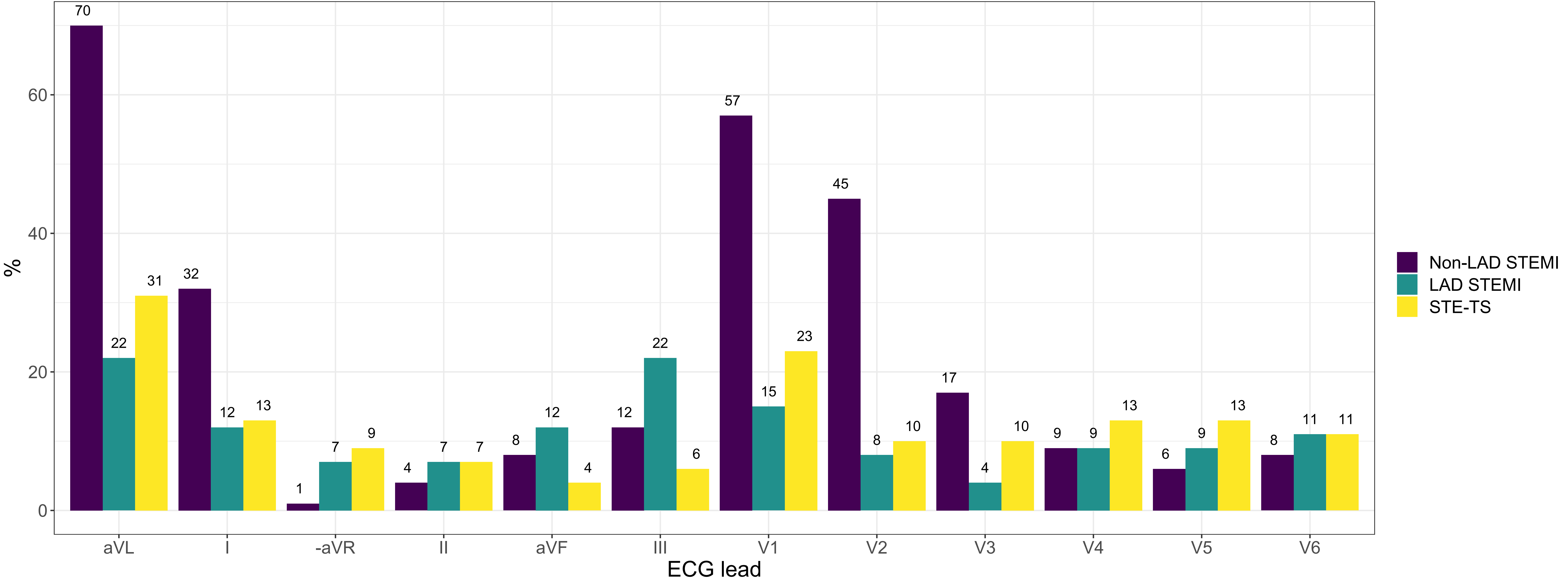

STEMI = ST elevation myocardial infarction; LAD = left anterior descending artery; STE-TS = ST elevation Takotsubo syndrome.
