# Supplementary material for: ECG differences and ECG predictors in patients presenting with ST segment elevation due to myocardial infarction versus takotsubo syndrome
Source: Int J Cardiol Heart Vasc. 2022 May 6;40:101047. doi: 10.1016/j.ijcha.2022.101047 (PMC9096129; doi:10.1016/j.ijcha.2022.101047)
Supplement: Supplementary Table 1 [file mmc3.docx]

Supplementary table 1. ECG and arrhythmia definitions

| Variable | Definition |
| --- | --- |
| *Basic parameters* |  |
| - Rhythm | Sinus, atrial fibrillation or flutter, AV-nodal or other |
| - Heart rate | Beats per minute |
| - PR-interval | Milliseconds |
| - QRS-duration | Milliseconds |
| - QRS axis | Degrees |
| - T-wave-axis | Degrees |
| - QTc-time | Milliseconds |
| - First-degree AV block | PR-interval > 220 milliseconds |
| - Second-degree AV-block |  |
| - - Mobitz I | Progressive prolongation of PR-interval before missing QRS |
| - - Mobitz II | PR-interval unchanged with intermittent missing QRS |
| - Third-degree AV-block | No association between P-waves and QRS |
| *Additional parameters* |  |
| - ST-elevation | ≥ 1 millimeter elevation of the ST-segment measured the at J-point from peak to the isoelectric line (nearest 0.5 millimeter) in any two anatomically consecutive leads |
| - ST-depression | ≥ 1 millimeter depression of the ST-segment measured 60 milliseconds after the J-point from nadir to the isoelectric line in any two anatomically consecutive leads |
| - ST-elevation with reciprocal ST-depression | ≥ 1 millimeter ST-segment elevation in two anatomically consecutive leads with ≥ 1 millimeter ST depression in two consecutive electrically opposite leads |
| - T-wave inversion | Negative T-wave with depth > 1 millimeter in any lead except for -aVR or V1 (-aVR and V1 included only in lead specific T-wave inversion) |
| - Q-wave pathology | Negative deflection preceding R-wave with duration > 40 ms or > 2 millimeters deep or > 25 % of QRS amplitude in two anatomically consecutive leads |
| - Long QTc | QTc duration > 440 milliseconds in males and > 460 milliseconds in females |
| - Fragmented QRS | Notching of R or S wave or the presence of more than one R’ in two contiguous leads |
| - Low voltage QRS | QRS complex with amplitude ≤5 millimeters in all limb leads or ≤ 10 millimeters in all precordial leads |
| - ST-elevation morphology | Concave or non-concave shape of the ST-segment as observed from the J-point from to the beginning of the T-wave |
| *Presenting ST-elevation pattern* | |
| - Anterior | ST-elevation ≥ 1 millimeter in V1-V2, V2-V3 or V3-V4 |
| - Lateral | ST-elevation ≥ 1 millimeter in V5-V6 or I-aVL |
| - Inferior | ST-elevation ≥ 1 millimeter in II-aVF or aVF-III |
| - Anterolateral | ST-elevation ≥ 1 millimeter in V1-V2, V2-V3 or V3-V4 and V5-V6 or I-aVL |
| - Inferolateral | ST-elevation ≥ 1 millimeter in II-aVF or aVF-III and V5-V6 or I-aVL |
| - Anterior-inferior | ST-elevation in V1-V2, V2-V3 or V3-V4 and II-aVF or aVF-III |
| - Anterior-inferior-lateral | ST-elevation in V1-V2, V2-V3 or V3-V4 and II-aVF or aVF-III and V5-V6 or I-aVL |
| - Other | ST-elevation pattern not fitting any of the above mentioned |
| *Arrhythmias* |  |
| - LTVA | Sustained VT or VF |
| - Sustained VT | VT with duration > 30 seconds or requiring cardioversion |
| - VT/VF | Any sustained or non-sustained VT or VF |
| - VT | Three or more ventricular extrasystoles at a rate of > 130 beats per minute |

LTVA = Life-threatening ventricular arrhythmia; VF = ventricular fibrillation; VT = ventricular tachycardia.
