# Supplementary material for: ECG differences and ECG predictors in patients presenting with ST segment elevation due to myocardial infarction versus takotsubo syndrome
Source: Int J Cardiol Heart Vasc. 2022 May 6;40:101047. doi: 10.1016/j.ijcha.2022.101047 (PMC9096129; doi:10.1016/j.ijcha.2022.101047)
Supplement: Supplementary Table 2 [file mmc4.docx]

Supplementary table 2. Lead specific ST elevation and ST depression in STE-TS vs LAD and non-LAD STEMI

|  | **STE** | **STD** | **STE** | **STD** | **STE** | **STD** | **STE** | **STD** | **STE** | **STD** | **STE** | **STD** |
| --- | --- | --- | --- | --- | --- | --- | --- | --- | --- | --- | --- | --- |
|  | *aVL* | | *I* | | *-aVR* | | *II* | | *aVF* | | *III* | |
| STE-TS | 14/104  (14%) | 2/104  (1.9%) | 17/104  (16%) | 2/104  (1.9%) | 20/104  (19%) | 0/104  (0%) | 27/104  (26%) | 3/104  (2.9%) | 22/104  (21%) | 3/104  (2.9%) | 22/104  (21%) | 6/104  (5.8%) |
| STEMI |  |  |  |  |  |  |  |  |  |  |  |  |
| LAD | 38/113  (34%) | 9/113  (8.0%) | 35/113  (31%) | 7/113  (6.2%) | 13/113  (12%) | 15/113  (13%) | 19/113  (8.8%) | 28/113  (25%) | 8/113  (7.1%) | 36/113  (32%) | 8/113  (7.1%) | 41/113  (36%) |
| Non-LAD | 7/161  (4.3%) | 120/161  (75%) | 6/161  (3.7%) | 68/161  (42%) | 44/161  (27%) | 2/161  (1.2%) | 116/161  (72%) | 2//161  1.2% | 147/161  (91%) | 5/161  (3.1%) | 145/161  (90%) | 6/161  (3.7%) |
| STE-TS vs… |  |  |  |  |  |  |  |  |  |  |  |  |
| LAD* | <0.001 | 0.043 | 0.012 | 0.17 | 0.11 | <0.001 | <0.001 | <0.001 | 0.0027 | <0.001 | 0.0027 | <0.001 |
| Non-LAD* | 0.0073 | <0.001 | <0.001 | <0.001 | 0.13 | 0.52 | <0.001 | 0.38 | <0.001 | >0.99 | <0.001 | 0.55 |
|  | *V1* | | *V2* | | *V3* | | *V4* | | *V5* | | *V6* | |
| STE-TS | 32/104  (31%) | 1/104  (1.0%) | 69/104  (66%) | 2/104  (1.9%) | 77/104  (74%) | 0/104  (0%) | 65/104  (63%) | 3/104  (2.9%) | 47/104  (45%) | 3/104  (2.9%) | 33/104  (32%) | 3/104  (2.9%) |
| STEMI |  | |  | |  | |  | |  | |  | |
| LAD | 64/112  (57%) | 2/112  (1.8%) | 100/112  (89%) | 1/112  (0.9%) | 91/111  (82%) | 4/111  (3.6%) | 68/111  (61%) | 9/111  (8.1%) | 36/112  (32%) | 16/112  (14%) | 15/112  (13%) | 15/112  (13%) |
| Non-LAD | 12/161  (7.5%) | 48/161  (30%) | 15/161  (9.3%) | 88/161  (55%) | 26/157  (17%) | 44/157  (28%) | 37/161  (23%) | 22/161  (14%) | 57/161  (35%) | 13/161  (8.1%) | 56/161  (35%) | 12/161  (7.5%) |
| STE-TS vs… |  |  |  |  |  |  |  |  |  |  |  |  |
| LAD* | <0.001 | >0.99 | <0.001 | 0.61 | 0.16 | 0.12 | 0.85 | 0.095 | 0.049 | 0.0031 | 0.0012 | 0.0052 |
| Non-LAD* | <0.001 | <0.001 | <0.001 | <0.001 | <0.001 | <0.001 | <0.001 | 0.0034 | 0.11 | 0.083 | 0.61 | 0.12 |

*P-value.

LAD = left anterior descending artery; STD = ST depression; STE = ST elevation; STEMI = ST elevation myocardial infarction; STE-TS = ST elevation Takotsubo Syndrome.
