# Supplementary material for: ECG differences and ECG predictors in patients presenting with ST segment elevation due to myocardial infarction versus takotsubo syndrome
Source: Int J Cardiol Heart Vasc. 2022 May 6;40:101047. doi: 10.1016/j.ijcha.2022.101047 (PMC9096129; doi:10.1016/j.ijcha.2022.101047)
Supplement: Supplementary Table 3 [file mmc5.docx]

Supplementary table 3. Predictors of VT/VF or death within 72 hours in patients with STEMI (LAD and non-LAD) and STE-TS

| Variable | LAD  N=113 | | Non-LAD  N=161 | | STE-TS  N=104 | |
| --- | --- | --- | --- | --- | --- | --- |
|  | OR (95%CI) | p-value | OR (95%CI) | p-value | OR (95%CI) | p-value |
| Sum of all ST-elevations |  |  |  |  |  |  |
| Univariable | 1.07 (1.01 – 1.14) | 0.026 | 1.08 (1.02 – 1.15) | 0.012 | 0.924 (0.779 - 1.10) | 0.37 |
| Model A* | 1.08 (1.01 – 1.15) | 0.016 | 1.08 (1.01 – 1.15) | 0.021 | 0.925 (0.777 - 1.10) | 0.38 |
| Model B† | 1.10 (1.02 – 1.19) | 0.0130 | 1.09 (1.02 – 1.16) | 0.0140 | 0.908 (0.750 - 1.10) | 0.32 |
| Sum of all ST-deviations |  |  |  |  |  |  |
| Univariable | 1.04 (0.992 – 1.09) | 0.10 | 1.08 (1.03 – 1.13) | 0.0012 | 0.915 (0.771 - 1.08) | 0.30 |
| Model A | 1.05 (0.998 – 1.10) | 0.062 | 1.08 (1.03 – 1.13) | 0.0020 | 0.916 (0.770 - 1.09) | 0.32 |
| Model B | 1.08 (1.01 – 1.15) | 0.021 | 1.08 (1.03 – 1.14) | 0.0016 | 0.896 (0.742 - 1.08) | 0.26 |
| Maximum single-lead ST-elevation |  |  |  |  |  |  |
| Univariable | 1.16 (0.936 – 1.43) | 0.18 | 1.65 (1.22 – 2.23) | 0.0013 | 0.656 (0.304 – 1.41) | 0.28 |
| Model A | 1.18 (0.952 – 1.47) | 0.13 | 1.65 (1.21 – 2.24) | 0.0016 | 0.657 (0.300 – 1.44) | 0.29 |
| Model B | 1.26 (0.977 – 1.61) | 0.076 | 1.66 (1.22 – 2.27) | 0.0014 | 0.637 (0.274 – 1.48) | 0.29 |
| ST-elevation with reciprocal ST-depression |  |  |  |  |  |  |
| Univariable | 1.91 (0.797 – 4.58) | 0.15 | 2.25 (1.19 – 4.24) | 0.013 | 2.36 (0.433 - 12.9) | 0.32 |
| Model A | 2.20 (0.887 – 5.43) | 0.089 | 2.47 (1.27 – 4.77) | 0.0074 | 2.31 (0.415 - 12.9) | 0.34 |
| Model B | 2.12 (0.826 – 5.46) | 0.12 | 2.46 (1.24 – 4.87) | 0.010 | 2.26 (0.369 - 13.8) | 0.38 |
| T wave inversion |  |  |  |  |  |  |
| Univariable | 0.929 (0.437 – 1.98) | 0.85 | 1.58 (0.624 – 4.01) | 0.33 | 0.133 (0.0163 - 1.08) | 0.059 |
| Model A | 0.921 (0.427 – 1.99) | 0.84 | 1.92 (0.725 – 5.09) | 0.19 | 0.118 (0.0142 - 0.988) | 0.049 |
| Model B | 0.947 (0.422 – 2.08) | 0.87 | 1.95 (0.688 – 5.50) | 0.21 | 0.132 (0.0154 – 1.13) | 0.064 |
| Long QTc‡ |  |  |  |  |  |  |
| Univariable | 0.527 (0.222 – 1.25) | 0.15 | 1.00 (0.461 – 2.19) | 0.99 | 0.133 (0.0163 – 1.08) | 0.059 |
| Model A | 0.573 (0.238 – 1.38) | 0.22 | 0.982 (0.439 – 2.20) | 0.96 | 0.126 (0.0151 – 1.05) | 0.055 |
| Model B | 0.389 (0.156 – 0.974) | 0.044 | 0.900 (0.385 – 2.11) | 0.81 | 0.115 (0.0128 – 1.04) | 0.054 |

*Adjusted for age and sex; †adjusted for age, sex, diabetes and previous myocardial infarction; ‡Long QTc >440 milliseconds for men, >460 milliseconds for women.

LAD = left anterior descending artery; LTVA = life threatening ventricular arrhythmia; STEMI = ST elevation myocardial infarction; STE-TS = ST elevation takotsubo syndrome, VT/VF = ventricular tachycardia or ventricular fibrillation.
